# Context specific challenges of the WHO infection prevention and control core components in the Faranah region: a mixed methods approach

**DOI:** 10.3389/fpubh.2025.1605425

**Published:** 2025-07-23

**Authors:** Anna Borodova, Amadou Aziz Diallo, Christine Timbo Songbono, Carlos Rocha, Ibrahima Nabé, Frederic Le Marcis, Mahamoud Sama Cherif, Sophie Alice Müller

**Affiliations:** ^1^Centre for International Health Protection, Robert Koch Institute, Berlin, Germany; ^2^Faranah Regional Hospital, Faranah, Guinea; ^3^Centre de Recherche et de Formation en Infectiologie de Guinée, Conakry, Guinea; ^4^TransVIHMI, University of Montpellier, Institut National de la Santé et de la Recherche Médicale (INSERM), Institut de Recherche pour le Développement (IRD), Montpellier, France; ^5^Direction Regionale de la Santé de Faranah, Ministère de la santé et de l’hygiène publique, Faranah, Guinea

**Keywords:** IPCAF, core component, hand hygiene, mixed method, healthcare workers, Guinea

## Abstract

Infection prevention and control (IPC) programs have been reported to reduce healthcare associated infections by up to 70%. These rates vary globally, with scare data suggesting that the highest prevalence occurs in the African region exceeding 50% in Guinea. The Infection Prevention and Control Assessment Framework facilitates the evaluation of WHO guidelines on core components of infection prevention and control programs. Using this framework, selected healthcare facilities in Faranah, Guinea were evaluated ahead of an implementation of a training of trainer’s program. Between May 2023 and March 2024, in a mixed method approach, the core components of 25 healthcare facilities were assessed in conjunction with an evaluation of 17 trainer’s knowledge and perception on hand hygiene through standardized tools. Findings were further triangulated through a deductive analysis consisting of participant observations and semi structured interviews. The overall median of the Infection Prevention and Control Assessment Framework score in the region was basic (242.5, IQR 172.5–342.5). Lowest scores were reported for IPC education, whereas IPC guidelines and healthcare associated infection surveillance received high scores. Rural healthcare centers had the lowest score (210.0, IQR 157.5–265.0), confirmed by qualitative assessment indicating a lack of allocated budget in these facilities in addition to generally observed patient and staff overload. Participant observation found that while healthcare associated infection surveillance scored highly and IPC guidelines were displayed on posters; their practical application was rare. This was triangulated with healthcare workers self-reporting hand hygiene compliance of up to 90% whereby demonstrating considerable gaps in knowledge of WHO hand hygiene standards. Our study provides detailed understanding of a resource limited setting and highlights the importance of continuous IPC training together with behavior changes and the improvement of healthcare associated infection surveillance. In settings where a majority reside in rural areas, appointed health centers must be paid special attention to as they may often be underserved. Finally, infrastructural challenges such as the allocation of budget, patient and staff overload need to be addressed in order to improve the health and safety of patients and healthcare workers.

## Introduction

Infection prevention and control (IPC) is an evidence-based approach to protect patients and healthcare workers (HCWs) from avoidable infections (1). Being responsible for excess mortality, long-term disability, additional financial burden and costs to government, patients and families, these healthcare associated infections (HAIs) are considered as a major health problem worldwide (2). The World Health Organization (WHO) places great importance in the reduction of HAIs and emphasizes the critical role of IPC, as up to 70% HAIs can be prevented through effective IPC interventions (3).

Globally, HAIs prevalence varies between 9.0 and 12.9%, while highest HAI prevalence of 27.0% is reported in the African region (4). Scarce data in this region suggest even higher prevalence rates of up to 54.2% in Guinea (5, 6). In Guinea, these high HAIs rates coincide with recurring outbreaks of Diphtheria (7), Yellow fever (8), Measles, Lassa, or Marburg virus disease (9) in a setting where only 36% of healthcare facilities meet at least 50% of the IPC minimum requirements (3). In order to combat this triple burden, numerous international actors have supported IPC programs, such as JHPEIGO, Expertise France and Robert Koch Institute (RKI) (10–12) and the Guinean Ministry of Health (MoH) has developed national guidelines for norms and procedures focusing on IPC (13).

The Infection Prevention and Control Assessment Framework (IPCAF) was created by the WHO in 2018 (14) in order to provide a baseline assessment of the IPC core components and promote their implementation. Global IPCAF surveys describe a score of 500.0 (IQR 345.0–657.5) (15), however no official Guinean survey has been published. Hand hygiene (HH) knowledge and perception has been previously assessed at the Faranah Regional Hospital (FRH), Guinea, as part of a mixed method study between December 2017 and August 2019. After an initial knowledge increase, a waning was reported, supporting the need for continuous trainings in IPC.

Following the IPCAF framework, the current study seeks to assess the IPC programs and activities at selected health facilities in the Faranah region in order to better identify the context specific challenges. With themes identified and deductively analyzed through qualitative approaches, these in-depth insights will allow for a better suited training approach in order to improve IPC knowledge and practice.

## Methods

### Study setting

The study was part of PASQUALE (Partnership to Improve Patient Safety and Quality of Care) (12), a partnership between the Faranah Regional Health Inspectorate (IRS), Guinea and the Robert Koch Institute (RKI) in Berlin, Germany, carried out in close collaboration with the Regional and Prefectural Health Directorate as well as the FRH.

The project takes place in the Faranah Region, a region with a population of around 1.2 million (16) bordering Sierra-Leone and Mali. Faranah receives an uneven allocation of healthcare provisions in comparison to the capital, has one of the lowest universal health coverage rates and the highest maternal and neonatal mortality rates in the country, making it a priority region (17). It comprises four prefectures (Dabola, Dinguiraye, Faranah and Kissidougou). Each prefecture has one hospital, whereby the only regional hospital is located in Faranah. The whole region encloses 49 healthcare centers (HCCs) and 207 healthcare posts (18).

Within the Guinean MoH IPC guidelines, HCWs are a targeted group in IPC improvement plans, since they are directly involved in patient care and are a source of HAI transmission (19). While improvement plans were developed within the country, many faced difficulties in implementation due to the lack of infrastructure, such as personal protective equipment or running water (19).

Established in 2017 at the FRH, PASQUALE first addressed the WHO Global Patient Safety Challenge: “Clean Care is Safer Care” (20). In 2023 during the third phase of the project, the focus expanded from hand hygiene to a holistic approach in IPC within all four prefectures of the region. In addition to all four prefectural hospitals, urban and rural HCCs were purposively selected on the regional level by each Prefectural Health Department (DPS). Inclusion criteria included accessibility, patient load and need for improvement. These selected healthcare facilities (HCFs) were invited to participate in the IPCAF (14).

A subset of those HCFs was included in the qualitative assessment supervised in Guinea at the Centre de Recherche et de Formation en Infectiologie de Guinée (CERFIG). One of the authors [CT], who was not previously known to participants, conducted a three-month ethnography between the months of May and December 2023.

In addition, a local training of trainer (ToT) program was established, in which one representative of the respective healthcare level from each prefecture (hospital, HCCs, private facilities, DPS), one representative from PASQUALE, and one representative from the IRS were invited to participate. All participants were purposively chosen by local authorities based on national criteria such as state employment, at least 10 years until retirement, and IPC responsibilities. All of those who did not meet this criterion were excluded. The training consisted of a 2-weeks on-site training on IPC theory and practice given by two national trainers. The training was framed by a pedagogical input on teaching methods for adults.

### Study design

This study is part of an overall mixed-methods research module for the assessment of IPC capacity in the Faranah region of Guinea, combining descriptive epidemiological and anthropological data, aiming at assessing IPC facility level, HCWs knowledge and attitudes as well as context-specific challenges to IPC program implementation. It followed predominately a parallel design (21) where the qualitative analysis of some of the qualitative data collected as part of the overall research responded to critical points identified by the quantitative data analysis.

### Quantitative

From May to August 2023, the research team visited all 25 selected HCFs and filled out the IPCAF (14) together with the administration of the respective HCF. The IPCAF is an international standardized WHO tool to assess the current IPC situation on facility level including IPC activities and resources. The tool can be used to identify strengths and shortcomings to inform future action plans.

The IPCAF is divided into eight sections reflecting the WHO IPC core components (CC), CC1: IPC program, CC2: IPC guidelines, CC3: IPC education, CC4: HAI surveillance, CC5: Multimodal strategies, CC6: Monitoring/audit of IPC practices and feedback, CC7: Workload, staffing and bed occupancy, CC8: Environments, materials and equipment for IPC. In total, the tool includes 81 indicators, whereby points are allocated to every indicator, depending on the importance of the question for the respective CC (14).

In March 2024, all 17 participants of the ToT were invited to fill in anonymized HH knowledge and attitude questionnaires before (baseline) and directly after (follow-up) the IPC training program. Anonymity of the questionnaires was incorporated to reduce social desirability and promote honest responses (22). These WHO questionnaires are standardized and previously validated (23). Each participant received an identification number that was used to pair HH knowledge and perception questionnaires before and after the training.

### Statistical analysis

Data was entered independently by two members of the research team into Microsoft Excel or Epi Info 7.2.3.0 and analyzed using STATA in version 17 (StataCorp. 2021. Stata Statistical Software: Release 17. College Station, TX: StataCorp LLC) (Table 1).

**Table 1:** Study population.

Characteristics	*n* (%)
Number of respondents	17
Gender: female	3 (17.7)
Age, median (IQR)	39 (36–42)
Profession	
Medical Doctor	8 (47.1)
Technician	6 (35.3)
Nurse	2 (11.8)
Other	1 (5.9)
Affiliation	
Hospital	5 (29.4)
HCC	4 (23.5)
DPS/IRS	5 (29.4)
Private facility	3 (17.7)
Additional characteristics	
Head of facility	6 (35.3)
Focal point IPC	9 (52.9)
Other	2 (11.8)

DPS, Prefectural Health Department; HCC, healthcare center; IPC, infection prevention and control; IQR, interquartile range; IRS, Regional Health Directorate.

For the IPCAF survey, we performed a descriptive analysis, aggregating the score of every CC, with a maximum of 100 points. The final IPCAF score was calculated by adding the scores of all CCs, equaling a maximum of 800 points. Based on the final score, HCFs were rated as inadequate (0–200 points), basic (201–400 points), intermediate (401–600 points) and advanced (601–800 points) (14). A detailed analysis of strengths and weaknesses per CC was given. Total and sub-scores were compared across HCF type by Kruskal Wallis test for overall comparison, followed by Conover-Iman test with Bonferroni correction for bivariate comparison.

With regards to the knowledge survey, frequencies and proportions of categorical response were summarized. Baseline and follow-up responses were compared in a paired analysis applying the Stuart-Maxwell Marginal homogeneity test.

HH perceptions were given either on a seven-point Likert scale (one “not effective,” seven “very effective”), as a percentage estimation or by a dichotomous question. For the Likert scale and percentage estimations, median and interquartile (IQR) estimates are given and compared with paired Wilcoxon signed-rank test between baseline and follow-up.

For all statistical tests a significant level of 0.05 was applied.

### Qualitative

During the three-month ethnography in parallel to the quantitative study, CT performed participant observations of daily hospital activities and had informal conversations with HCWs, patients and families in three of the selected HCFs, including a hospital, a rural and an urban HCC. Across the whole data collection period, CT kept a fieldwork journal where she provided a thorough account of the daily IPC practices and emerging topics vis-à-vis her overall research objective which is the materialization of IPC recommendations and guidelines in the hospital routines. Semi-structured interviews with 13 informants identified as key by the analysis of the fieldwork journal were conducted with the inclusion of one group discussion. Topic guides were developed according to CT’s own literature research, with emergent topics identified on the fieldwork journal and further aimed at triangulating the data already collected. Other members of the qualitative team (FLM and CR) supervised the data collection by reading fieldwork reports to provide a timely feedback and enhance CT’s reflexivity on emerging topics and redirection of fieldwork due to possible biases and assumptions.

For the purposes of this paper, critical points identified in the IPCAF score (e.g., low scoring of rural HCCs) were provided to the qualitative research team. This resulted in the creation of a codebook to conduct a deductive analysis for more in-depth understanding on these critical points within the collected qualitative data.

Fieldwork, transcription, and analysis were primarily conducted in French, occasionally in *Kissie* and *Maninkakan*; verbatim quotations were translated into French as needed, and into English by the authors of this manuscript. Coding using Nvivo was performed.

### Ethical approval and consent to participate

The PASQUALE 3 project is currently operating in all four prefectures (Dabola, Dinguiraye, Kissidougou, Faranah) in Faranah, Guinea under the ethical approval granted by *Le Comité National d’Éthique pour la Recherche en Santé* (198/CNERS/23), in Conakry Guinea. All participants in the HH questionnaires and qualitative part gave written consent to be included in the study. Concerning the IPCAF, all observed HCFs are in the framework of the project which include health authorities at the regional and prefectural level. No informed consent was needed since information collected did not concern sensitive patient data but only general information on IPC in HCFs. Oral confirmation was requested and deemed sufficient from respective authorities.

## Results

### Quantitative

#### Study sites

In total, the IPCAF was applied to 25 HCFs, of which the Kissidougou Prefectural Hospital was the largest in terms of bed and staff capacity and FRH in terms of monthly consultations (Table 2). Comparing rural HCCs, highest consultations numbers were reported in Dabola.

**Table 2:** Characteristics of included HCFs.

HCFs by prefecture		Total number of beds	Total number of personnel (contractual and volunteers)	Total number of cleaners	Mean of monthly consultations (07/23–09/23)
Dabola					
	Hospital	60	99	18	895
	1 Urban HCC	6	38	2	1,061
	3 Rural HCCs	27	105	20	6,002
Dinguiraye					
	Hospital	50	124	14	915
	1 Urban HCC	3	50	0	1,553
	3 Rural HCCs	18	70	5	5,313
Faranah					
	Hospital	80	125	17	7,195
	3 Urban HCCs	16	55	6	2,372
	4 Rural HCCs	39	102	8	2,811
Kissidougou					
	Hospital	122	148	25	2011
	3 Urban HCCs	25	229	12	2,158
	3 Rural HCCs	47	76	35	916

HCC, healthcare center; HCF, healthcare facility.

#### IPCAF

The overall median score was 242.5 (IQR 172.5–342.5), corresponding to a basic IPC level. When grouped into IPC level, eight HCFs (32.0%) fell into the category “inadequate,” 15 (60.0%) into “basic,” and two hospitals (8.0%) into “advanced” (FRH: 622.5/800.0; Kissidougou Prefectural Hospital 627.5/800.0, respectively). Looking at IPC status by HCF level, rural HCCs had significantly lower scores than hospitals (Table 3).

**Table 3:** IPCAF Score in selected HCFs of the Faranah region.

Prefectures and HCFs		Number of HCFs assessed	IPCAF score median (IQR)	IPC level	*p*-value
Overall scores		25	242.5 (172.5–342.5)	Basic	
Prefecture					
	Dabola	5	180.0 (172.5–240.0)	Inadequate	0.091
	Dinguiraye	5	155.0 (115.0–157.5)	Inadequate	Ref.
	Faranah	8	270.0 (212.5–398.8)	Basic	0.009
	Kissidougou	7	340.0 (265.0–380.0)	Basic	0.002
Health facility level					
	Hospitals	4	510.0 (343.8–625.0)	Intermediate	0.003
	Urban HCCs	8	271.3 (191.3–360.0)	Basic	0.167
	Rural HCCs	13	210.0 (157.5–265.0)	Basic	Ref.

HCC, healthcare center; HCF, healthcare facility; IPC, infection prevention and control.

IPC Level: Inadequate < 201, Basic ≥ 201, Intermediate ≥ 401, Advanced ≥ 601.

**Figure 1:**
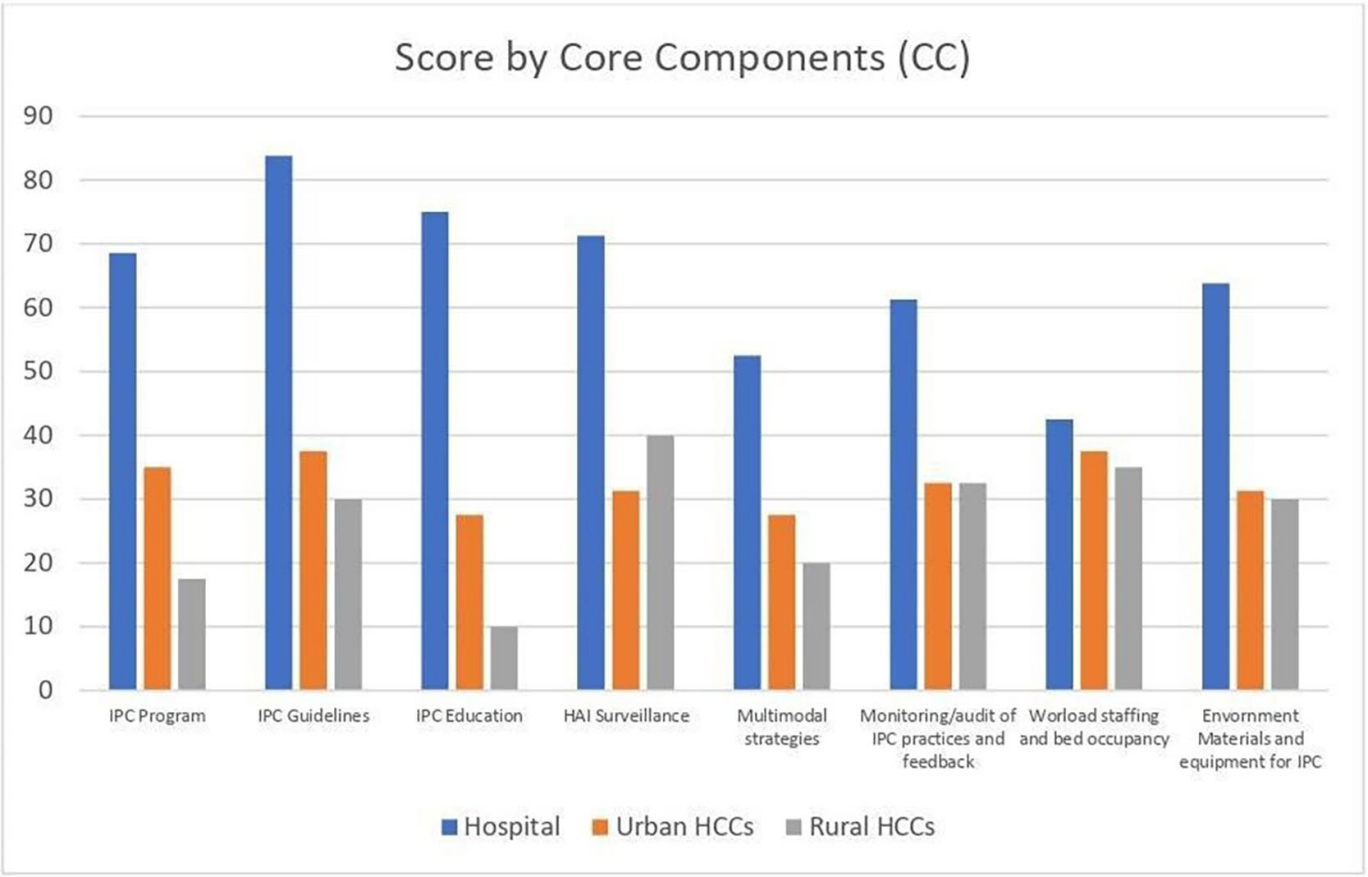
Score by core components (CC).

In a more detailed account, all CC had a lower score in rural HCCs, as seen in Table 4 and Figure 1. Highest CC scores were tied in the region for IPC guidelines 40.0 (IQR 20.0–60.0) and HAI surveillance 40.0 (IQR 27.5–57.5). IPC education had the lowest score in the region with 15.0 (IQR 10.0–35.0), whereas IPC program 20.0 (IQR 7.5–42.5), and multimodal strategies 20.0 (IQR 5.0–35.0) were tied as second lowest. IPC program, guidelines and education were all statistically significantly lower in Urban HCCs and Rural HCCs in reference to Hospitals in the region.

**Table 4:** Score by core component (CC).

Core components^#^	Total median (IQR)	Hospital median (IQR)	Urban HCC median (IQR)	Rural HCC median (IQR)	*p*-value‘
CC1:	20.0 (7.5–42.5)	68.8 (46.3–78.8)	35.0 (3.8–45.0)	17.5 (5.0–20.0)	0.015
		Ref.	0.018*	0.001*	
CC2:	40.0 (20.0–60.0)	83.8 (61.3–87.5)	37.5 (13.8–56.3)	30.0 (15.0–45.0)	0.044
		Ref.	0.015*	0.006*	
CC3:	15.0 (10.0–35.0)	75.0 (65.0–80.0)	27.5 (20.0–37.5)	10.0 (5.0–15.0)	<0.001
		Ref.	0.009*	<0.001*	
CC4:	40.0 (27.5–57.5)	71.3 (40.0–92.5)	31.3 (20.0–60.0)	40.0 (30.0–42.5)	0.290
CC5:	20.0 (5.0–35.0)	52.5 (17.5–77.5)	27.5 (0.0–40.0)	20.0 (10.0–20.0)	0.345
CC6:	32.5 (20.0–52.5)	61.3 (36.3–85.0)	32.5 (20.0–61.3)	32.5 (17.5–37.5)	0.133
CC7:	35.0 (30.0–50.0)	42.5 (25.0–60.0)	37.5 (25.0–50.0)	35.0 (35.0–45.0)	0.906
CC8:	32.5 (27.5–52.5)	63.8 (45.0–71.3)	31.3 (23.8–47.5)	30.0 (22.5–37.5)	0.049
		Ref.	0.015*	0.007*	

HCC, healthcare center; HCF, healthcare facility; IPC, infection prevention and control; IPCAF, Infection Prevention and Control Assessment Framework; IQR, interquartile range.

^#^CC1: IPC program, CC2: IPC guidelines, CC3: IPC education, CC4: HAI surveillance, CC5: Multimodal strategies, CC6: Monitoring/audit of IPC practices and feedback, CC7: Workload, staffing and bed occupancy, CC8: Environments, materials and equipment for IPC.

‘Using Kruskal Wallis Test *applying Conover-Iman test with Bonferroni correction if Kruskal Wallis for overall comparison significant.

When looking at CC1, only 12/25 HCFs (48.0%) had an IPC program (3 rural HCCs, 5 urban HCCs, 4 hospitals), whereby only 6/22 (27.3%) (1 rural HCC, 2 urban HCCs, 3 hospitals) had an active IPC comity. 12/20 (60.0%) of HCFs had a microbiology laboratory for routine use, but only 7/12 (58.3%) provide reliable results. There was no documented microbiological laboratory in any of the hospitals.

In terms of CC2, a minority reported to have the “Expertise for developing and adapting national guidelines” (4/9, 44.4%) or “Specific trainings on updated IPC guidelines” (4/20, 20.0%).

In CC3, the majority (17/23, 73.9%) of HCFs had no regular IPC training for HCWs in place. No HCFs provided IPC training for patient or their families.

When looking at CC4 and surveillance activities, in 21/25 (84.0%) of HCFs, surveillance was part of the IPC program and in CC5, 4/21 HCFs (19.1%) use “Multimodal strategies to implement IPC interventions.”

In regards to CC6, 5/25 HCFs (20.0%) had no monitoring indicators at all, but the majority monitored HH compliance, consumption of alcohol-based handrub (ABHR) or waste management (17/25, 68%; 15/25, 60.0%; 16/25, 64.0%; respectively).

In CC7, 20/23 HCFs (87.0%) reported inappropriate staffing levels based on national standards, including 75% of the hospitals. 17/24 HCFs (70.8%) documented having more than one patient per bed in certain departments, while 4/24 (16.7%) mentioned a regular overcharge.

Finally, in CC8, 14/23 HCFs (60.9%) had insufficient access to running water, whereby 7/25 (28.0%) had stable access to drinking water and reliable electricity supply. 20/24 HCFs (83.3%) reported no individual rooms to cohort patients with similar pathogens, including 50% of included of hospitals. In total, a vast minority of HCFs (4/25, 16.0%) stated to have a functional incinerator.

#### Trainer survey

In total, 17 questionnaires on HH knowledge and perception were collected before (baseline) and after (follow-up) the ToT. The participants were predominately medical doctors (8, 47.1%), and/or focal points of IPC (9, 52.9%) in the respective HCFs.

##### Knowledge

Table 5 displays selected knowledge questions of interest from the 17 participants.

**Table 5:** Selected training participant responses to knowledge questions, *n* (%), *N* = 17.

Selected knowledge questions	Preferred response‘	Baseline	Follow-up
What is the most frequent route of cross transmission?	HCW’s hands	8 (47.1)	13 (76.5)*
What is the minimal time needed for ABHR to kill most germs on your hands?	20 s	7 (41.2)	12 (70.6)*
Which type of HH method is required in the following situations? - after removing examination gloves	Rubbing with ABHR	8 (47.1)	6 (35.3)*
Which type of HH method is required in the following situations? - after emptying a bedpan	Rubbing with ABHR	1 (5.9)	2 (11.8)*
Which type of HH method is required in the following situations? - before giving an injection	Rubbing with ABHR	11 (64.7)	9 (52.9)*
Which type of HH method is required in the following situations? - after visible exposure to blood	Handwashing	14 (82.4)	16 (94.1)*
Handrubbing causes skin dryness more than handwashing	False	7 (41.2)	6 (35.3)*
Handrubbing is more effective against germs than handwashing	True	8 (47.1)	8 (47.1)*
Handrubbing is more rapid for hand cleansing than handwashing	True	16 (94.1)	17 (100.0)*

‘, responses reported as correct by the WHO.

*, differences not significant between baseline and follow-up based on Stuart-Maxwell Marginal homogeneity test.

ABHR, Alcohol-based handrub; HH, hand hygiene.

Participants improved their knowledge on route of transmission and minimal time of ABHR use considerably. The questions on “required method of hand hygiene” showed mixed results; whereby the vast majority (94.1%) of participants were aware of the necessity for handwashing after “visible exposure to blood,” only 11.8% reported rubbing with ABHR as the correct method after “emptying a bedpan.” Knowledge on the required method “after removing examination gloves” and “before giving an injection” declined across assessment rounds (47.1 to 35.3%, 64.7 to 52.9%, respectively). While all participants in follow-up rated handrubbing to be more rapid than handwashing, 35.3% believed that handrubbing was causing more skin-dryness than handwashing and 47.1% estimated handrubbing to be more effective than handwashing.

##### Perception

Table 6 displays selected perception questions. All participants rated HH to be effective in preventing HAIs (follow-up) and estimated that between 30.0% (baseline) and 15.0% (follow-up) of hospitalized patients will develop a HAI.

**Table 6:** Selected training participant responses to perception questions, median (IQR), *n*.

Selected perception questions	Baseline	Follow up
Is the use of ABHR well tolerated by your hands?	NA	7.0 (7.0–7.0), *n* = 17
… what is the average percentage of hospitalized patients who will develop a health care-associated infection?	30.0 (20.0–40.0), *n* = 9	15.0 (5.0–17.5), *n* = 12*
… in what percentage of situations requiring HH do HCWs (…) actually perform HH, either by handrubbing or handwashing?	60.0 (50.0–80.0), *n* = 10	80.0 (70.0–100.0), *n* = 14*
… in what percentage of situations requiring HH do you actually perform HH, either by handrubbing or handwashing?	80.0 (60.0–90.0), *n* = 17	75.0 (60.0–90.0), *n* = 13*
How effective would the following actions be? - Patients are invited to remind HCWs to perform HH	6.0 (3.0–7.0), *n* = 17	6.0 (4.0–7.0), *n* = 16*
How effective would the following actions be? -Leaders and senior managers at your institution support and openly promote HH	6.0 (5.0–7.0), *n* = 17	7.0 (7.0–7.0), *n* = 17
How effective would the following actions be? - The health-care facility makes ABHR always available at each point of care	6.0 (5.0–7.0), *n* = 17	7.0 (6.0–7.0), *n* = 16*
How effective would the following actions be? - HH posters are displayed at point of care as reminders	7.0 (5.0–7.0), *n* = 17	7.0 (6.0–7.0), *n* = 16*
How effective would the following actions be? - Each HCW receives education on HH	6.0 (4.0–7.0), *n* = 17	6.0 (5.0–7.0), *n* = 16*
How effective would the following actions be? - Clear and simple instructions for HH are made visible for every HCW	7.0 (6.0–7.0), *n* = 17	6.5 (5.0–7.0), *n* = 16*
How effective would the following actions be? - HCWs regularly receive feedback on their HH performance	6.0 (3.0–7.0), *n* = 17	7.0 (5.0–7.0), *n* = 16*
How effective would the following actions be? - You always perform HH as recommended (being a good example for your colleagues)	6.0 (5.0–7.0), *n* = 17	7.0 (6.0–7.0), *n* = 16*

*, differences not significant between baseline and follow-up based on Wilcoxon signed-rank test homogeneity test.

ABHR, alcohol-based handrub; HCW, Healthcare worker; HH, hand hygiene, NA, not applicable.

All participants reported to routinely use ABHR throughout the study, and generally gave high estimates of compliance to HH among themselves [baseline: 80.0 (IQR: 60.0–90.0); follow-up 75.0 (60.0–90.0]). 82.4% felt that ABHR was very well tolerated by their hands.

When speaking about HH interventions, 15/17 (88.2%) considered leaders and managers at their facility to be supportive. In addition, when rating different aspects of the multimodal WHO HH campaign, the inclusion of patients to remind HCWs of HH as well as available ABHR, posters, education, instructions, performance feedback on HH an being a good example were rated as (very) effective.

### Qualitative

Qualitative observations through participant observations and personnel interview among key personnel, including hygienists, nurses, midwives, medical doctors, surgeons, technical health assistants, and laboratory technicians have addressed three key areas.

(1) IPC strengths of the [name of town] prefecture and hospital:

The main points that were reported during the qualitative interviews and observations were ownership in conjunction with motivation.

One hospital director insisted on the importance of reducing HAI rates.

*“I consider the [name of town] hospital to be my own home, since my parents attend there. My aim is to offer quality care to my people to reduce the rate of nosocomial infections and deaths. I really do not want hospital staff to give them [patients] other illnesses.”* Interview, May 2024.

This motivation was transferred to the hospital staff in terms of involvement in decision making. The hygiene committee, created and led by the hospital staff, ensured patient safety and further developed IPC interventions. Regarding ownership, these interventions were continuously monitored and evaluated by hospital staff on a monthly basis across all departments. The results of these internal evaluations were then used to inform authorities of potential adjustments to enhance good clinical practice.

In addition, the administration has implemented a system to overcome gaps or delays in state funding. This system was also used to pay performance incentives for hospital staff to show recognition and sustain motivation. In addition, as a common practice in the Guinean health system (24), the management of the hospital allocated an additional lump sum that varied according to the revenue generated by the hospital each month.

(2) Low IPCAF scores of rural HCCs:

Qualitative research showed that unlike hospitals, rural HCCs did not receive state subsidies, but operated on donations and the income generated by the care provided. Within the framework of IPC, there was no allocated budget, making it a lower priority when procuring supplies for rural HCCs.

*“The supply of IPC inputs to the health centers is based on donations […] As for the gloves, they have not been coming for a very long time. Since I’ve been here, there have not been any kits. Since February 2023, gloves are included in the kits. The DPS has no budget for the purchase of IPC inputs for the health centers. Either the centers receive donations or they buy with their own income.”* Interview, June 2024.

This lack of allocated budget led to an unsustainable supply in personal protective equipment, such as examination gloves. Given the high prices of those materials, some higher than medicine, (priority in purchasing was given to essential therapeutics).

*“Now about the glove issue (…). But you know when the Ministry sends products for reproductive health, if there are gloves there, that’s what’s convenient, but if there aren’t, the centers buy those gloves. These gloves are even more expensive than the medicines.” Interview, June 2024.*

*“One day there’s a center that calls that it does not have a glove. But if I take my consumption in gloves, I calculate that, I will not even be able to buy other medicines.” Interview, June 2024.*

(3) General recommendations:

In terms of general recommendations, the local research team reported, that the IPCAF tool should optimally be applied by independent researchers, since the presence of donors or policy representatives could create bias, such as social desirability bias. The IPCAF assessment itself should be performed on a regular basis, as direct changes after an assessment might be put into practice. Which was the case in our study, as an IPC department was created directly after our visit at one prefectural hospital.

During the observations, a wide range of international IPC guideline posters were found in the targeted structures. These posters, featuring the logos from the Guinean Ministry of Health or from donors, such as USAID, WHO or Expertise France, were generally displayed on walls of different services and wards, witnessing the history of the deployment and health governance of IPC in Guinea. Qualitative observation of the clinical practices yielded a rare influence of these guidelines on IPC practice, emphasizing the need for inclusion of local HCWs such as medical doctors, nurses and directors in guideline development order to maximize applicability and feasibility.

Observations of hospital routines also confirmed a general lack of post-Ebola IPC training, with a specific need for administrative and management staff. A fact that was also emphasized within the IPCAF assessment (Table 4).

Finally, although crucial component of IPC, surveillance of HAIs was difficult to translate into medical practice in the targeted HCFs. Only a small number of HCFs collected data on germs with epidemic potential or snakebites. Even if laboratory capacities were in place, there was a lack of clinical identification systems and clinical practitioners rarely sent their patients for assessments given the extra financial burden to the patient.

## Discussion

This study gives a detailed overview of structural and clinical IPC in a low-resource setting, such as the Faranah region in Guinea. As part of an overall mixed-method research for the assessment of IPC capacity combining descriptive epidemiological and anthropological data, we aimed to identify gaps and strengths to inform local authorities and policy makers on need based and applicable interventions to sustainably improve IPC in an epidemic-prone setting. Using internationally recognized tools and working alongside local authorities, our study captures in-depth practices of the selected HCFs in the region of Faranah.

### IPCAF

We evaluated 25 HCFs in the IPCAF assessment, including hospitals, urban and rural HCCs across all four prefectures of the Faranah region. Overall, the region showed a basic IPC score, with two prefectures having inadequate scores, and with higher scores for hospitals than rural HCCs. The overall score of the region and of all individual prefectures ranked generally lower than reported in the 2022 WHO global survey for the African region (15), and lower than the score reported for Low Income Countries in the 2024 global report (3). Nevertheless, two hospitals of the region demonstrated advanced scores outscoring other hospitals in low resource settings, such as Sierra Leone or Uganda by 250 to 300 points (25, 26).

Overall, HCFs showed highest scores in CC2 (IPC guidelines) and CC4 (HAI surveillance), comparable to a mixed-method study on two other sub-Saharan African HCFs (27). This is contrary to the WHO Global IPC Report, where HAI surveillance is one of the least developed pillars reported in the African region, for which 24.3% of countries have a national strategic plan (3). The high scores on HAI surveillance are questioned by the lack of laboratory capacities, as no hospital in the entire region provided a microbiological laboratory. A finding in line with recent data from low-resource settings, where only 1.0% are formally assigned to deliver bacterial testing (28).

Our study found differences in quantitative and qualitative findings, emphasizing the need for triangulation to ensure an in-depth understanding of IPC practices. For instance, the high scores in CC2 and CC4 of the IPCAF are in contradiction with the qualitative results as both IPC practice triggered by the presence of IPC guidelines and HAI surveillance were rarely observed. This pinpoints the difference between what is of theoretic importance for IPC, in particular for the hospital management who replied to these assessments, and what is practiced during hospital routines. This inconsistency highlights the challenges of implementing and integrating international guidelines into daily hospital routines. For example, the diagnostics and laboratory capacities for HAI surveillance can be in place, but their high costs for patients make their application difficult and are therefore rarely used for medical decision-making. These discrepancies could also be attributed to social desirability when administering the IPCAF, which was handled in person and usually with higher-ranking personnel. Overall, this mixed method approach is the main strength of our study as it can provide essential observations in contexts where HCWs or administration do not want to portray their healthcare facilities in a negative light, and will carry on throughout our continued research.

WHO’s first international evidence-based guidelines on the CC of IPC programs was published in 2016, in which continuous trainings including multimodal strategies to achieve behavioral changes have been confirmed as a pillar in IPC improvement (29). However, CC3 (IPC education and training) showed the lowest score, confirmed as well through qualitative results whereby post-Ebola, IPC training has not been a priority in the region. Our study reported considerable gaps in IPC improvement, which can be associated to limited access to qualified and trained IPC professionals and overall scarce evidence in implementing these guidelines in low resource settings, such as Faranah (29). For instance, the close second lowest CC in our study was CC5 (Multimodal strategies), with only 16.0% of HCFs using multimodal strategies to implement IPC surveillance and only 12.0% having an IPC program with clear objectives and responsibility. The majority of HCFs has no regular IPC trainings in place similar with data from other comparable settings such as Ghana (30). Overall, this absence of regular IPC trainings can also be due to the dependence on external interventions, since the vast majority of included HCFs lack an IPC committee. These IPC committees are dependent on staff motivation, which can be hard to upkeep when HCFs sometimes go without any state funding, and must priorities other hospital activities, as observed through qualitative interviews.

The FRH as the reference center of the region, has one of the highest numbers of consultations, although with a smaller bed capacity compared to other prefectural hospitals, such as Kissidougou our study confirms a widespread burden, with most HCFs reporting inadequate staffing and patient overload, where multiple patients regularly share a bed. This finding is in alignment with other data from Guinea, where only 4.43 skilled HCWs per 10,000 were reported (31), in comparison to the WHO needed standard of 23 HCWs per 10,000 (32). This existing staff shortage is further exacerbated by a continuing decline in medical personnel in the Faranah region (18). The Guinean health system hence heavily relies on medical volunteers, otherwise called “stagiaires” that are not contracted by the hospital or state, but rely on informal payments organized with health structures (33). HCWs who are formally hired may never or rarely come to work at an HCF, especially in rural areas further away from the capital of Conakry, while still receiving salary an official salary according to the civil servant registry (34). This absenteeism at work with rates up to 41.0% was confirmed in two regions of Guinea (34).

In our study, rural HCCs also see a high number of consultations. However, they report the lowest score on all components in comparison to other levels, which stresses the lack of attention and resources received despite high patient load. This observation was reinforced by qualitative research, which highlighted that budget constraints make it nearly impossible to maintain adequate supplies. As a result, HCWs use examination gloves only when deemed necessary.

WASH (Water, sanitation and hygiene) is the epitome of IPC (35). However, across our study sites, basic IPC infrastructure such as provision of incinerators, running water or safe drinking water were limited and ranked lower compared to the WHO global survey (15). The local ABHR production as introduced by the PASQUALE project can be a cornerstone to overcome this gap while working on general infrastructure improvement (36).

### HH knowledge

In conjunction with the lack of regular IPC trainings found in the IPCAF assessment, considerable gaps in baseline and follow-up knowledge were reported, particularly regarding the distinction of indications for handwashing and handrubbing. Handrubbing is the correct action for indicators such as “after removing examination gloves,” and “before giving an injection,” in which more participants answered incorrectly in the follow-up, choosing handwashing. Throughout both assessment periods, “after emptying a bedpan” had the lowest score with participants favoring handwashing. While handrubbing is considered the key to HH (2) and is promoted by national standards in Guinea (13), the absence of a framework for regular and continuous training of HCWs in low-resource settings could explain the knowledge gap in our study. In addition, as reported by the study team, missing regular update of national standardized operational guidelines for the different HH methods as well as their promotion and dissemination to the operational level can be mentioned as aggravating factors. Furthermore, a slight minority of participants estimated handrubbing to be more effective in killing germs than washing, a potentially worrisome result, when considering that our study participants will be future trainers of local HCWs. In conjunction with limited WASH possibilities, more awareness and promotion of the advantages of rubbing with ABHR is needed.

### HH perception

Participants showed high awareness of the prevalence of HAIs, potentially reflecting the high motivation to become an IPC trainer. This high motivation and awareness can be seen as the first and foremost step for behavioral change (37). While self-reported HH compliance was very high (up to 90%), previous HH observations in these settings showed considerably lower compliance rates between 45.1 and 75.1% (38, 39). This discrepancy underlines potential room for improvement and need to refine self-evaluation. A similar discrepancy was observed regarding the perceived and actual reported rates of HAIs. A potential for improvement and sensibilization was also noted when asking participants about ABHR usage. While most participants reported in perception questionnaires to routinely use ABHR, in knowledge questionnaires participants tended to prefer handwashing over rubbing and also reported side effects such as skin dryness due to ABHR. With two ABHR production sites in the region, there are increasing opportunities to overcome WASH challenges by promoting ABHR as an effective tool for HH. In general, participants rated implementation steps such as posters, positive examples as well as inclusion of patients as very effective. These recommended implementation steps can be helpful for policy makers, but need to be seen in the local context with literary rates of around 45.3% in Faranah (40).

### Limitations

Our study has several limitations. To enhance reliability of the assessed data, the IPCAF assessment was done with the local study team and not through self-evaluation, since the region struggles with proper internet, electricity and is not fully digitized. However, the presence of the local study team could have led to certain social desirability bias that could inflate reported practices, highlighting the importance of triangulation, the strength of our mixed-method approach. In future studies, to further reduce this bias, broader facility sampling or independent assessments are recommended. Furthermore, we used the detailed version of the IPCAF, which was sometimes difficult to apply to the rural HCCs of these resource limited settings. If the recently published HCFs specific and minimized IPCAF (41) could give similar detailed results, can be assessed in future project activities. Qualitative observations found contradictory results to our quantitative assessments, highlighting the need to request documentation and background information for each of the eight IPCAF core components, such as HAI surveillance, as a means to triangulate social desirability from the facilities’ governance during further assessments.

The HCFs as well as training participants were purposively chosen, limiting the generalizability of our results while assuring that all types of HCFs and the entire region are represented. The small number of training participants could also lead to constraints in conclusion about knowledge gains and perceptions of hand hygiene in their respective health facilities. Also, because of the reachability, there were no trainers from rural HCCs, however the DPS were among the study participants as representatives of all HCCs.

The anonymized HH questionnaire was not directly linked to the IPC training and hence does not completely reflect the acquired knowledge, but uses the previously standardized WHO questionnaires (42) to enable international comparison. Furthermore, our study team noticed, that some questions such as those on the distinction between the protection of HCWs and patients were hardly understood, reflecting the need for guided application of adapted questionnaires in applied research.

## Conclusion

In this epidemic prone, but resource limited setting, our study found low levels of IPC in both HCF staff and structures. In addition to lacking WASH infrastructure, IPC programs and multimodal strategies, the absence of regular training led to gaps in knowledge and the preference of handwashing over rubbing. We recommend not only continuous IPC training, but also continuous assessments with the IPCAF across health facilities in order to guide policy makers in recommendations and monitor progress over time especially in areas where there is lack of surveillance data on the WHO core components. Further investing in essential WASH improvements and procurement of basic personal protective equipment, such as gloves, can be also a first step in improving IPC preparedness.

Our study finds it is essential that structural and personnel challenges receive the same level of attention, as well as to align the limited available resources to the respective patient charge especially in marginalized rural settings. For sustainable and reliable outcomes in IPC preparedness, these recommendations should be supported and monitored by internal and local structures such as IPC and hygiene committees.

## Data Availability

The original contributions presented in the study are included in the article/supplementary material, further inquiries can be directed to the corresponding author.

## Glossary

ABHR: Alcohol- based handrub
CC: IPCAF Core components
CERFIG: Centre de Recherche et de Formation en Infectiologie de Guinée
DPS: Prefectural Health Department
FRH: Faranah Regional Hospital
HAIs: Healthcare- associated- infections
HCW: Healthcare worker
HCFs: Healthcare facilities
HCCs: Healthcare centers
HH: Hand Hygiene
IPC: Infection prevention and control
IPCAF: Infection prevention and control assessment framework
IQR: Interquartile range
IRS: Regional Health Directorate
MoH: Ministry of Health
NA: Not applicable
PASQUALE: Partnership to improve Patient Safety and Quality of Care
ToT: Training of the Trainer
WHO: World Health Organization
WASH: Water, sanitation and hygiene
